# From growth and fixed creative mindsets to creative thinking: an investigation of the mediating role of creativity motivation

**DOI:** 10.3389/fpsyg.2024.1353271

**Published:** 2024-06-20

**Authors:** Wu-jing He, Tin-wai Chiang

**Affiliations:** Department of Special Education & Counselling, The Education University of Hong Kong, Tai Po, Hong Kong SAR, China

**Keywords:** creativity, growth creative mindset, fixed creative mindset, creativity motivation, implicit theories of creativity

## Abstract

Studies documenting and seeking to understand the mindset effect have yielded mixed and inconclusive findings. The present study sought to address the research question pertaining to the mindset effect on creative thinking and its underlying mechanism from the perspectives of social cognitive theory and mindset theory, which postulate a motivational mechanism underlying the mindset-creativity link. Specifically, this study aimed to examine the mediating role of creativity motivation in the effects of growth and fixed creative mindsets on creative thinking. A convenience sample of 948 college students from three universities in Hong Kong participated in the study. Creative mindset, creativity motivation, and creative thinking were assessed using the Chinese version of the Creative Mindset Scale, the Creativity Motivation Scale, and the Test for Creative Thinking-Drawing Production (TCT–DP), respectively. Lending support to the perspectives of social cognitive and mindset theories, the results of mediation analyses conducted using Preacher and Hayes’s bootstrapping approach indicated that creativity motivation had partial mediating effects on the positive and negative roles of growth and fixed mindsets, respectively, in creative thinking. Enriching the research on the motivation mechanism underlying the impacts of creative mindsets on creative thinking, the results further illustrated that creativity motivation has a stronger mediating effect on the impact of growth creative mindset on creative thinking than on that of fixed creative mindset. The possible theoretical and educational implications of the findings of this research are discussed.

## 1 Introduction

Creativity, commonly defined as the production of an idea or product that is novel and useful (Sternberg et al., 2024), has long been an important research topic due to its significant contribution to personal success and societal progress. In recent years, research on implicit theories of creativity, conceptualized in terms of laypeople’s inner beliefs regarding creativity, have been emerging and flourishing (Karwowski, 2023). Notably, an increasing research attention has been paid to a subset of implicit theories of creativity (i.e., *creative mindset*), which specifically pertains to people’s beliefs regarding the stable-versus-malleable nature of creativity (Emami et al., 2023; Hua and Yang, 2024). Joining this line of research, the present study aimed to understand the effect of two types of creative mindset (i.e., *growth and fixed creative mindsets*) on creative thinking and the associated underlying mechanism that may account for this effect. More precisely, the study sought to examine the effect of growth and fixed creative mindsets on creative thinking via the mediating role of creativity motivation from the perspectives of *social cognitive theory* and *mindset theory*.

### 1.1 The perspective of social cognitive theory

According to social cognitive theory (Bandura, 1977, 2023), learning occurs in a social context with a dynamic and reciprocal interaction of the person, environment, and behavior. Linking to implicit theories, the theory suggests that people’s inner beliefs about abilities are learned from the interaction of person, environment, and behaviors (Abedini et al., 2023). It further highlights that people’s inner beliefs function as general principles for directing and influencing intentional behaviors, and people accept their inner beliefs as true regardless of evidentiary support, which impacts their motivational tendencies and, subsequently, outcome behaviors (Rad et al., 2023; Hamann et al., 2024). Taking this perspective, researchers postulated that implicit theories function as antecedents of task motivation, which in turn determines the direction and intensity of the effort made to accomplish a goal (Beauchamp et al., 2019; Zheng et al., 2023). In other words, this theoretical perspective postulates a motivational mechanism of inner beliefs, which emphasizes that task motivation plays a mediating role in connecting implicit theories of abilities (i.e., predicting variables) and outcome behaviors (i.e., dependent variables).

Applying social cognitive theory in the context of creativity, researchers have suggested that relevant creativity beliefs (i.e., implicit theories of creativity) may play a facilitating (or inhibiting) role in creativity motivation, thereby leading to enhanced (or reduced) creativity-related behavioral tendencies (e.g., Li et al., 2021; Sternberg and Karami, 2021; Emami et al., 2023; Lin and Wang, 2023). For example, researchers built on social cognitive theory and proposed the Creative Behavior as Agentic Action (CBAA) model to explore the motivational function of creativity beliefs on creative outcomes (Karwowski and Beghetto, 2019; Karwowski et al., 2020; Beghetto and Karwowski, 2023; Karwowski, 2023). In particular, the CBAA model contends that people’s creative actions are largely influenced by their motivational tendencies, which result from their inner beliefs regarding creativity (He, 2022; Beghetto and Karwowski, 2023; Zielińska et al., 2023, 2024; Ivcevic et al., 2024). In other words, the CBAA model represents the perspective of social cognitive theory by theoretically postulating that implicit theories of creativity impact creative outcomes via the mediation of creativity motivation.

### 1.2 The perspective of mindset theory

Creative mindset, a subset of implicit theories of creativity (Karwowski, 2014), concerns specifically people’s inner beliefs regarding the source and dynamic nature of creativity, i.e., whether creativity is an inborn and unchangeable ability or a malleable and developable skill (Karwowski, 2023). In particular, researchers have built on the mindset theory of intelligence (Dweck, 1986, 2006; Dweck and Yeager, 2020) by proposing two types of creative mindset, namely, *growth and fixed creative mindsets*. Growth creative mindset, endorsed by incremental theorists, refers to the belief that creativity is a changeable skill that can be trained and nurtured through efforts and practices. Fixed creative mindset, on the contrary, is endorsed by entity theorists and refers to the beliefs that creativity is innate and fixed and cannot be changed or developed no matter how much effort or practice one invests (see also Beghetto and Karwowski, 2023; Karwowski, 2023; Zielińska et al., 2023, 2024; Ivcevic et al., 2024).

Regarding the motivational function of these two types of creative mindset, growth creative mindset is proposed to play a facilitating role, whereas fixed creative mindset is alleged to play an inhibiting role (Beghetto and Karwowski, 2023; Karwowski, 2023; Yeh et al., 2023). For example, Zhou et al. (2020) suggested that people who exhibit a growth creative mindset are more motivated to engage in a creative process that involves multiple trials and errors when attempting to accomplish a creative task because they believe in the effectiveness of motivated effort with regard to creative pursuits. In contrast, people who exhibit a fixed mindset do not believe that motivated effort is effective with regard to changing creative outcomes due to the unchangeable nature of creativity. As a result, such people are less motivated to engage and invest effort in a creative task, consequently reducing their likelihood of achieving promising creative outcomes. Other researchers (e.g., Li et al., 2021; Doss and Bloom, 2023; Emami et al., 2023; Yeh et al., 2023) have made similar claims, proposing that people with a growth creative mindset are more motivated to engage in creative endeavors and creative interventions due to their belief that engagement and sustained effort can change the corresponding outcomes. In contrast, people with a fixed creative mindset tend to see no reason to engage in any such creative endeavors or creative interventions due to their belief in the unchangeable nature of creativity. In summary, these views are in line with social cognitive theory by postulating that creativity motivation (i.e., mediators) plays a mediating role in the relationship between creative mindsets (i.e., predicting variables) and creative outcomes (dependent variables). This perspective anticipates a facilitating role of growth creative mindset in creative outcomes through enhanced creativity motivation, whereas an inhibiting role of fixed creative mindset in creative outcomes through reduced creativity motivation.

### 1.3 Empirical findings and the present study

Despite the theoretical claims about the growth and fixed mindset effects, empirical studies seeking to understand these effects produced mixed findings in various performance domains (e.g., academic achievement and well-being; Sisk et al., 2018; Bui et al., 2023; Burnette et al., 2023; Lou and Li, 2023; Macnamara and Burgoyne, 2023), leaving the empirical examination of the mindset effects an open and inconclusive research question. In the context of creativity, empirical research exploring the mindset effects on creative performance and the expected motivational mechanism has just emerged in recent years (Li et al., 2021; Yeh et al., 2023). In particular, several empirical studies have examined the motivational function of growth creative mindset and found initial empirical support for the positive role of growth creative mindset in various motivational aspects of creative behaviors (e.g., Karwowski and Brzeski et al., 2017; Zhang et al., 2018; Yeh et al., 2023). For example, it has been found that people who exhibited a growth creative mindset were more motivated to engage in creativity training, perform creative activities, and participate in creative hobbies. Moreover, when people’s growth creative mindset led to an increased level of motivation, it indirectly contributed to enhanced explicit creative behaviors (Karwowski and Brzeski, 2017). In a recent study, Li et al. (2021) directly examined the mediation of creativity motivation in the link between growth creative mindset and creative achievements and found empirical support for the anticipated role of creativity motivation. Specifically, Li et al.’s (2021) findings illustrated that when participants showed a higher level of growth creative mindset, they also exhibited a higher level of creativity motivation, which in turn led to more creative achievements. Similarly, Yeh et al. (2023) also found supporting evidence which illustrated that growth creative mindset functioned as a significant motivational variable that positively predicted enhanced creative self-efficacy in game-based creativity learning.

With respect to fixed creative mindset, although some research findings have lent support to the theoretical claims regarding its detrimental effect on creative outcomes (e.g., Royston and Reiter-Palmon, 2017; Yodchai et al., 2022), relatively fewer empirical works have directly addressed the research question of whether such a negative effect is attributable to the mediating role of a reduction in creativity motivation. In this context, it is interesting to note that some indirect empirical findings have revealed a mixed pattern regarding the mediating role of creativity motivation on the relationship between fixed creative mindset and creative outcomes, with some such studies reporting supportive evidence and other such studies failing to do so. For example, Intasao and Hao (2018) observed that fixed creative mindset inhibited creative performance in an insight problem task by reducing task enjoyment and task effort, in which context task enjoyment and task effort were regarded as proxy measures of intrinsic motivation. However, in another study, Zhou et al. (2020) failed to replicate these results regarding the significant mediating effect of task effort in the context of the negative role of fixed creative mindset in supervisor-rated creativity, although they did find that task effort significantly mediated the effect of the growth creative mindset on creativity. Similarly, Yeh et al. (2023) also failed to find supporting evidence for the anticipated motivational mechanism in relation to the inhibitory effect of fixed mindset by illustrating that fixed creative mindset did not show any significant influence on the motivational variable (i.e., self-determination) or the creative outcome variable (i.e., creative self-efficacy).

In summary, the limited research findings available in the literature suggest that empirical testing of theoretical claims regarding the motivational role of creative mindset in creativity is still in its infancy, and the findings that have been reported in this context remain far from conclusive. While most of these empirical works have focused primarily on growth creative mindset, the motivational impact of fixed creative mindset on creativity remains a relatively under researched topic. Moreover, although initial empirical evidence has been found to support the anticipated mediating role of creativity motivation in the effect of growth creative mindset, research findings have revealed a mixed pattern regarding the effect of fixed creative mindset. Hence, the empirical question regarding the mediating role of creativity motivation in the relationship between such creative mindsets (especially fixed creative mindset) and creative outcomes remains unresolved, and the main objective of the present study was to address this research question. Drawing on the theoretical perspectives of social cognitive theory and the mindset theory of creativity, the current study aimed to verify the respective facilitating and inhibiting roles of the growth and fixed creative mindsets in creative thinking through the mediation of enhanced or reduced creativity motivation. Specifically, the following two hypotheses are tested:

*Hypothesis 1*: Growth creative mindset facilitates creative thinking through the mediation of enhanced creativity motivation.

*Hypothesis 2*: Fixed creative mindset inhibits creative thinking through the mediation of reduced creativity motivation.

## 2 Methods

### 2.1 Participants and procedures

A cross-sectional study was conducted across three universities in Hong Kong. Undergraduate and postgraduate students studying arts, education, engineering, social sciences, sciences, and healthcare related discipline participated in this study. The inclusion criteria were: (1) undergraduate and postgraduate students aged 18–26 years, (2) having no apparent physical or psychological disability (students diagnosed with mental disorders or under treatment in the recent 6 months were excluded), (3) having no problems in understanding verbal and written Chinese (students indicating difficulties in reading and comprehending Chinese were excluded), and (4) having consented to participate.

A convenience sampling method was used because of its advantages of cost effectiveness and ready availability to the researchers. To minimize possible sampling bias, a random sampling technique was used to allow an equal chance for each eligible student to participate. In this regard, an open invitation to participate was sent with the aim of reaching out to all eligible students via (1) public recruitment advertisements on campuses, (2) mass email invitations on intranets, and (3) sharing links on social media. Interested students were invited to attend a debriefing session to gain more understanding of the nature of the study and their expected involvement in the study. However, they were blinded to the specific study questions and hypotheses to avoid potential expectancy bias. Only those students who provided signed written informed consent were invited to participate in the study, and all participate was voluntary. The final sample consists of 948 (52.1% females) undergraduate and postgraduate students who had a mean age of 21.4 years (*SD* = 1.96; range = 18–25 years) and an average education level of 15.2 years (*SD* = 1.83; range = 12–17 years). All participants were ethnic Chinese and spoke Cantonese as their mother language. Table 1 summarizes the demographic characteristics of the sample. The statistics of the demographic characteristics such as parents’ education level and monthly household income suggest that the participants were mainly from middle-class or lower-middle-class socioeconomic backgrounds. Specifically, the mothers and fathers had an average education level of 13.8 (*SD* = 2.09; equivalent to a high school degree) and 15.0 years (*SD* = 3.11; equivalent to a bachelor’s degree), respectively. Regarding family income, 17.2% of the participants reported a low-level monthly household income (i.e., less than HK$20,000), 67.5% reported a medium-level income (i.e., between HK$20,001 and HK$40,000), while 15.3% reported a high-level income (i.e., more than HK$40,001; Census and Statistics Department, 2023).

**Table 1:** Demographic characteristics of the sample (*n* = 948).

Characteristics	Mean	*SD*
Age (years)	21.4	1.96
Education (years)	15.2	1.83
Mother’s education (years)	13.8	2.09
Father’s education (years)	15.0	3.11

Characteristics	Frequency	%
Gender		
Male	454	47.9
Female	494	52.1
Academic year		
Year 1	153	16.1
Year 2	186	19.6
Year 3	184	19.4
Year 4	178	18.8
Year 5	139	14.7
Master	108	11.4
Monthly household income (HK$)		
Less than $10,000	20	2.11
$10,001 – $20,000	144	15.1
$20,001 – $30,000	309	32.6
$30,001 – $40,000	331	34.9
$40,001 – $50,000	101	10.7
More than $50,000	43	4.54

The median monthly household income in Hong Kong was HK$30,000 during the year of data Research Topic (Census and Statistics Department, 2023).

Data collection was conducted by experienced research staff who were blinded to the study aims and research hypotheses. Assessments of the study variables (i.e., creative mindset, creativity motivation, and creative thinking) were administered to the participants alongside standard instructions in a group setting that featured approximately 20–25 participants. Prior to data collection, all participants were informed about the confidentiality, safety, and voluntary principles of the study, and their right to withdraw from the study at any time for any reasons without penalty consequences. The assessment procedure took approximately 35–40 min to complete.

### 2.2 Instruments

#### 2.2.1 Creative mindset

To assess creative mindset, the 10-item Creative Mindset Scale (Karwowski, 2014) was adapted and translated into Chinese using a back-translation procedure. The scale was developed as a two-factor structure to assess the two dimensions of creative mindset (i.e., growth and fixed creative mindset; Karwowski et al., 2019; Beghetto and Karwowski, 2023). Each of the growth and fixed creative mindset subscales consists of five items. A sample item measuring growth mindset is “Rome wasn’t built in a day– each creativity requires effort and work, and these two are more important than talent,” while a sample item measuring fixed mindset is “A truly creative talent is innate and constant throughout one’s entire life.” Responses are provided on a 5-point scale ranging from 1 (definitely no) to 5 (definitely yes). The scale’s psychometric properties and applicability with regard to a Chinese student sample were well supported by good confirmatory factor analysis (CFA) indices (CFI = 0.96, TLI = 0.95, RMSEA = 0.058, 90% CI = [0.031, 0.082], SRMR = 0.054; see Zhou et al., 2020).

In the current study, the obtained fit indices of a CFA also lent support to the two-factor model of the scale (*χ*^2^ = 67.4, *df* = 34, *χ*^2^/df = 1.98, CFI = 0.963, TLI = 0.958, RMSEA = 0.048, SRMR = 0.072). Furthermore, the convergent validity of the scale was assessed using the average variance extracted (AVE) and the composite reliability (CR), while the discriminant validity was assessed using the HeteroTrait-MonoTrait ratio of correlations (HTMT). The results revealed that an AVE value greater than 0.50 and a CR value greater than 0.70 were obtained for both the growth (AVE = 0.62; CR = 0.89) and fixed mindset scales (AVE = 0.57; CR = 0.87), confirming the convergent validity of the scales. Moreover, the obtained HTMT value (i.e., 0.21) was smaller than 0.90, lending support to the discriminant validity of the scale (Hair et al., 2021). With regard to reliability, *Cronbach’s α* (*α* = 0.85 for growth creative mindset; *α* = 0.82 for fixed creative mindset) and *McDonald’s ω* coefficients (*ω* = 0.83 for growth creative mindset; *ω* = 0.81 for fixed creative mindset) greater than 0.70 were obtained, supporting good internal consistency (Hayes et al., 2020).

#### 2.2.2 Creativity motivation

Creativity motivation was assessed using the 9-item Chinese version of the Creativity Motivation Scale (Zhang et al., 2018). This scale was developed based on creativity motivation theory, which conceptualizes creativity motivation as the motivational force that drives individuals to engage in creative activities such as learning, doing, and accomplishing new things (Li et al., 2021). A sample item related to learning new things is “It is useful to discover new things that I have never seen before.” A sample item related to doing new things is “It is important to do something in my own original way.” A sample item related to accomplishing new things is “I experience pleasure when I bring a perceptible product to completion.” Responses are provided on a 6-point scale ranging from 1 (strongly disagree) to 6 (strongly agree), thus indicating the extent to which participants agreed or disagreed with these statements. In previous research, the psychometric properties of the scale and its applicability among Chinese students in Hong Kong have been well supported, and evidence has been found to support its construct validity based on the fitness indices of CFA (CFI = 0.964, RMSEA = 0.055). Moreover, its convergent validity was supported by the average variance extracted (AVE; 0.51) and composite reliability (CR; 0.95) statistics, which were greater than the 0.50 and 0.70 thresholds, respectively. The internal consistency of the scale was also proven to be sufficient with Cronbach’s *α* = 0.83. In this study, the results of CFA suggest that the one-factor model have good fit indices (*χ*^2^ = 59.8, df = 27, *χ*^2^/df = 2.21, CFI = 0.965, TLI = 0.962, RMSEA = 0.046, SRMR = 0.039), confirming its construct validity. Besides, the results with respect to a calculated AVE value greater than 0.50 (i.e., 0.54) and a calculated CR value greater than 0.70 (i.e., 0.88) also supported the convergent validity of the scale. Moreover, the high *Cronbach’s α* (*α* = 0.86) and *ω scores* (*ω* = 0.84) obtained in this study also supported the internal reliability of the scale in the present sample.

#### 2.2.3 Creative thinking

Creative thinking was assessed using the Chinese version of the Test for Creative Thinking–Drawing Production (TCT–DP; Form A, Urban and Jellen, 1996; He and Wong, 2011), which was developed based on a gestalt approach to assess the ability of creatively combining unrelated components to produce a product that is evaluated to have the characteristics of novelty and usefulness (He, 2023b). In particular, the TCT–DP assesses creative thinking through a drawing task performed on an A4-sized testing sheet that contains six unrelated fragments: (a) a point, (b) a 90^o^ angle, (c) a curved line, (d) a broken line, (e) a semicircle, and (f) a small open square. Based on the gestalt approach, the drawing can be completed using any combination of the six fragments in a wide variety of ways, ranging from simple, conventional, and disjointed combinations to thematically complex, unconventional, integrated, and aesthetically interesting combinations. Creative thinking was scored according to nine criteria as stipulated by the TCT–DP test manual [i.e., Continuations, Completion, Connections by line, Connections by theme, New elements, Perspective, Humor and affectivity, Boundary breaking (which consists of 2 subcriteria), and Unconventionality (which consists of 4 subcriteria)]. The total possible score range is 0–66 points, with a higher composite score indicating a higher level of creative thinking. Numerous studies have reported evidence supporting the psychometric properties of the test as well as its applicability with regard to Chinese student samples (e.g., Rudowicz, 2004; He and Wong, 2011; He and Wong, 2022b; He, 2023b). For example, the validity of the instrument has been supported by its significant correlations with a wide range of well-established creativity measures pertaining to different aspects of creativity, such as divergent thinking, creative achievement, and creative personality (Rudowicz, 2004; He, 2018). Moreover, sufficient internal consistency of the test has also been reported (e.g., *α* = 0.89 in He, 2023b; *α* = 0.80–0.84 in He and Wong, 2022b). In this sample, reasonably good internal consistency (i.e., *α* = 0.83, *ω* = 0.81) was also obtained. Moreover, the results of CFA showed that the one-factor model had reasonably good fit indices (*χ*^2^ = 95.2, df = 32, *χ*^2^/df = 2.78, CFI = 0.904, TLI = 0.901, RMSEA = 0.055, SRMR = 0.042). Furthermore, the calculated AVE (i.e., 0.51) and CR (i.e., 0.95) were greater than 0.50 and 0.70, respectively, confirming the convergent validity of the scale.

### 2.3 Data analysis

The data analysis was conducted using the Statistical Package for Social Science (SPSS) software program version 28.0. Analysis was two-tailed, with a *p*-value of 0.05. In the first step of data analysis, descriptive analyses of all study variables were performed prior to testing the hypotheses to determine the normality of the data. Moreover, as all measurements were self-reported. There exists a potential confounding factor of common method variance. As a precautionary measure, the Harman’s single-factor test was used to detect the presence of common method variance. Subsequently in the second step of data analyses, Pearson correlation analysis was performed to determine whether the anticipated bivariate correlation could be found among the main study variables (i.e., creative mindsets, creativity motivation, and creative thinking). Lastly, in the third step, the hypotheses regarding the relationships among growth creative mindset and fixed creative mindset as independent variables (IVs), creative thinking as a dependent variable (DV), and creativity motivation as a mediating variable (Mediator) were assessed based on a mediation approach involving the bootstrapping method by using Hayes SPSS Process Macro (Preacher and Hayes, 2008). Specifically, Model 4 was tested with respect to the hypothesized simple mediation model regarding the underlying mechanism of creativity motivation (Mediator) on the effect of growth and fixed mindsets (IVs) and creative thinking (DV). In the mediation analysis, the 95% confidence intervals (CIs) of the indirect effects were obtained by reference to 5,000 bootstrap samples. The indirect effect was considered to be significant when the 95% CI did not include 0.

## 3 Results

### 3.1 Preliminary analyses

The results of the normality test suggested that all study variables were within the range of normal distribution, with skewness = −0.39–0.47 and kurtosis = 0.58–0.71 (Shanthi, 2019). The results of the Harman’s single-factor test further indicate that the potentially biasing factor owing to common method variance was minor in this sample because the first factor in the exploratory factor analysis only accounted for 32% of the overall load, which falls below the conventional threshold of 40% (Tehseen et al., 2017).

### 3.2 Bivariate correlations

Descriptive statistics and the correlation matrix of the study variables are presented in Table 2. Related to Hypothesis 1 (H1), which proposed that growth creative mindset plays a positive role in creativity motivation and creative thinking, the results of the correlation coefficients revealed that growth creative mindset was positively correlated with scores on both the creativity motivation test (*r* = 0.43, *p* < 0.001) and the TCT–DP (*r* = 0.36, *p* < 0.001) at a statistically significant level. In relation to Hypothesis (H2), which proposed that fixed creative mindset plays a negative role in creativity motivation and creative thinking, significant results were also found regarding the negative correlations between fixed creative mindset and the scores on both the creativity motivation test (*r* = −0.21, *p* < 0.01) and the TCT–DP (*r* = −0.27, *p* < 0.01). Furthermore, in accordance with both Hypotheses 1 and 2 regarding the positive role of creativity motivation in creative thinking, creativity motivation was found to be positively associated with the TCT–DP score at a statistically significant level (*r* = 0.51, *p* < 0.001).

### 3.3 Mediation analyses

**Figure 1:**
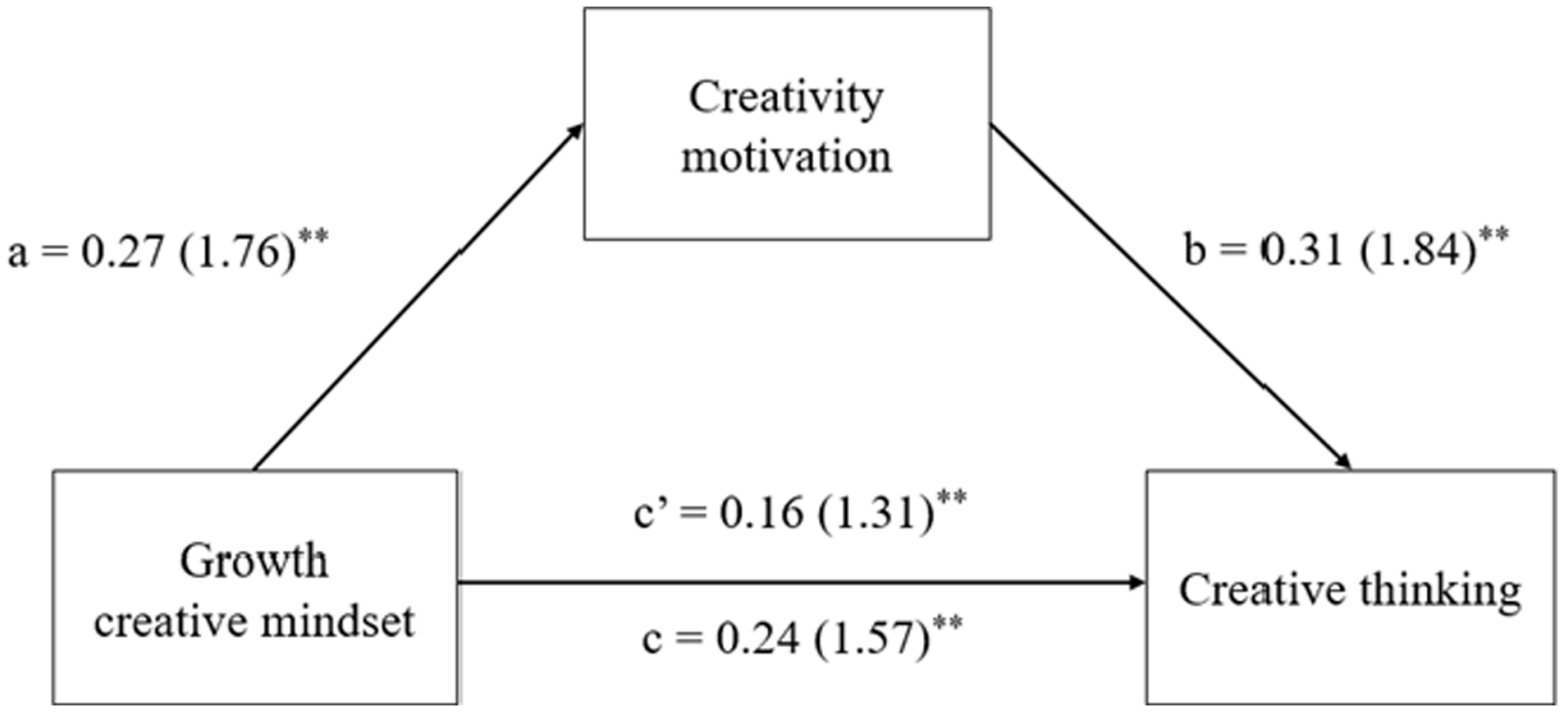
Results of mediation analysis for growth creative mindset, creativity motivation, and creative functioning. Standardized path coefficient are shown, with corresponding unstandardized coefficients in parentheses. ^*^p < 0.05, ^**^p < 0.01.

**Figure 2:**
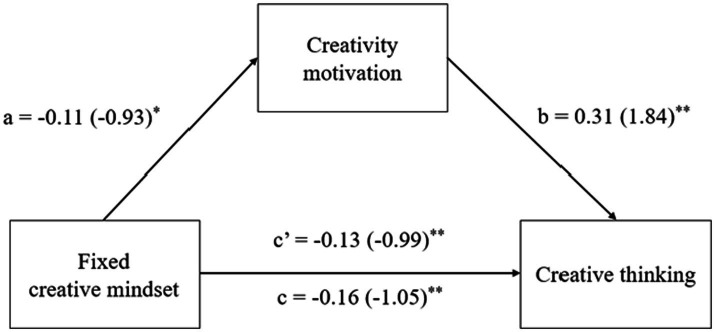
Results of mediation analysis for fixed creative mindset, creativity motivation, and creative functioning. Standardized path coefficient are shown, with corresponding unstandardized coefficients in parentheses. ^*^p < 0.05, ^**^p < 0.01.

To test the two hypotheses concerning the positive and negative impacts of growth and fixed creative mindsets (IVs), respectively, on creative thinking (DV) through the mediation of creativity motivation (Mediator), mediation analyses were conducted to investigate two separate mediation models, i.e., one for each independent variable (i.e., growth or fixed creative mindset). Relevant results are displayed in Table 3. See also Figures 1, 2 for a diagrammatic representation of the mediation models.

**Table 2:** Means, standard deviations (SD), Cronbach’s alpha coefficients (α), and correlation coefficients of the study variables.

	Mean (*SD*)	*α*	1	2	3	4
1. Growth creative mindset	3.11 (1.17)	0.85^**^	1.00	−0.12^*^	0.43^***^	0.36^***^
2. Fixed creative mindset	3.09 (1.45)	0.82^**^		1.00	−0.21^**^	−0.27^**^
3. Creativity motivation	3.14 (1.08)	0.86^**^			1.00	0.51^***^
4. TCT–DP	21.3 (3.98)	0.83^**^				1.00

TCT–DP, test for creative thinking–drawing production; ^*^p < 0.05, ^**^p < 0.01, ^***^p < 0.001.

Regarding H1, which predicted that a growth creative mindset would facilitate creative thinking through the mediation of enhanced creativity motivation, the results regarding the path coefficients shown in Table 3 and Figure 1 revealed that growth creative mindset (IV) had significant positive effects on both the DV (i.e., creative thinking; *c* = 0.24, *t* = 4.91, *p* < 0.01) and the mediator (i.e., creativity motivation; *a* = 0.27, *t* = 5.31, *p* < 0.01). In addition, the mediator (i.e., creativity motivation) was found to have a significantly positive impact on the DV (i.e., creative thinking; *b* = 0.31, *t* = 5.93, *p* < 0.01). In support of H1, the results regarding the indirect effect found by reference to 5,000 bootstrap samples indicated a significant indirect relationship between growth creative mindset and creative thinking, which was mediated by creativity motivation (a*b = 0.07, *t* = 2.31, *p* < 0.05), and the 95% CI did not include 0 (CI [0.06, 0.11]). These results further suggest that creativity motivation has a partial mediating effect on the impact of growth creative mindset on creative thinking because the direct effect of growth creative mindset on creative thinking was still found to be significant after controlling for the indirect effect of creativity motivation (*c’* = 0.16, *t* = 4.11, *p* < 0.01). Specifically, the results indicated that creativity motivation accounted for approximately 33.3% of the total effect of growth creative mindset on creative thinking [*P*_M_ = (0.08)/(0.24)*100%].

With respect to H2, which predicted that fixed creative mindset would hinder creative thinking through the mediation of inhibited creativity motivation, the results presented in Table 3 and Figure 2 revealed that fixed creative mindset had significant negative effects on both creative thinking (*c* = −0.16, *t* = −4.18, *p* < 0.01) and creativity motivation (*a* = −0.11, *t* = −3.14, *p* < 0.05), whereas creativity motivation had a significant positive effect on creative thinking (*b* = 0.31, *t* = 5.93, *p* < 0.01). With respect to the indirect effect, the results found by reference to 5,000 bootstrap samples lent support to H2 and revealed that creativity motivation had a significant mediating effect on the relationship between fixed creative mindset and creative thinking (a*b = 0.03, *t* = −2.01, *p* < 0.05), and the confidence interval did not include 0 (CI [−0.06, −0.01]). These results further suggest that creativity motivation has a partial mediating effect because the direct impact of fixed creative mindset on creative thinking was significant after controlling for the indirect effect of creativity motivation (*c’* = 0.13, *t* = 3.46, *p* < 0.05). Specifically, the results indicated that creativity motivation accounted for approximately 18.8% of the total effect of fixed creative mindset on creative thinking [*P*_M_ = (0.03)/(0.16)*100%] (Table 3).

**Table 3:** Results of mediation analyses.

	Bootstrap Estimate		95% CI	
Path/effect	*β*	*SE*	Bootstrap with bias correction	*P*_M_
*c* (Growth creative mindset ➔ TCT–DP)	0.24^**^	0.109	[0.12, 0.28]	33.3%
*a* (Growth creative mindset ➔ Creativity Motivation)	0.27^**^	0.113	[0.13, 0.30]	
*b* (Creativity Motivation ➔ TCT–DP)	0.31^**^	0.128	[0.11, 0.41]	
*c’*	0.16^*^	0.074	[0.05, 0.20]	
*a* x *b*	0.08^*^	0.002	[0.06, 0.11]	
*c* (Fixed creative mindset ➔ TCT–DP)	−0.16^**^	0.104	[−0.14, −0.31]	18.8%
*a* (Fixed creative mindset ➔ Creativity Motivation)	−0.11^**^	0.096	[−0.03, −0.28]	
*b* (Creativity Motivation ➔ TCT–DP)	0.31^**^	0.128	[0.11, 0.41]	
*c’*	−0.13^*^	0.073	[−0.04, −0.18]	
*a* x *b*	−0.03^*^	0.003	[−0.06, −0.01]	

TCT–DP, test for creative thinking–drawing production; *c*, total effect; *c’*, direct effect; *a* x *b*, indirect effect; 95% CI, 95% Confidence Interval; *P*_M_, proportion of the total effect for which the mediator accounts. ^**^p < 0.01, ^*^p < 0.05.

## 4 Discussion

Implicit theories of creativity have been linked to creative performance (Karwowski, 2023). Creative mindset, a subset of implicit theories of creativity, has received increasing research attention in recent years (Emami et al., 2023; Hua and Yang, 2024). Based on the frameworks of social cognitive theory and mindset theory, this study examined the mediation of creativity motivation in the impacts of both the growth and fixed creative mindsets on creative thinking. The findings thus obtained contribute to the literature by offering empirical evidence to support the theoretical claim with respect to the mediating role of creativity motivation in the impacts of both the growth and fixed creative mindsets on creative outcomes. These findings also illuminate the extent to which creativity motivation can account for the respective positive and negative effects of the growth and fixed creative mindsets on creative thinking, respectively. Highlights regarding the key findings of this study are presented below, followed by a discussion of the possible theoretical and educational implications of the findings of this research.

### 4.1 The mediating role of creativity motivation

Lending support to H1, the first major finding of the study confirmed that creativity motivation plays a significant partial mediating role in the positive effect of growth creative mindset on enhanced creative thinking. These findings are in alignment with several past behavioral studies that support a positive role of growth mindset in the motivational function of creative behaviors (e.g., Intasao and Hao, 2018; Zhang et al., 2018). These findings are also in agreement with neuroscientific evidence, which suggest a positive link between growth mindset and intrinsic motivation (Ng, 2018). These findings join Li et al. (2021) and Yeh et al. (2023) to provide direct empirical support for the theoretical claim regarding the motivational mechanism underlying the impacts of growth creative mindset on creative outcomes. Moreover, by estimating the extent (i.e., 33.3%) to which creativity motivation, as a mediator, was able to account for the impact of growth creative mindset on creative outcomes, the present research further enrich the mindset-motivation-creativity literature by adding new empirical evidence regarding the strength of impact of creativity motivation on the effects of growth creative mindset on creative thinking.

In relation to H2, the second major finding of the current study confirmed that creativity motivation plays a significant partial mediating role in the negative effect of fixed creative mindset on inhibited creative thinking. While some previous studies have reported evidence suggesting that fixed creative mindset has a negative impact on creative thinking (e.g., Intasao and Hao, 2018; Yodchai et al., 2022), limited empirical evidence has been reported regarding the mediation of creativity motivation in such a negative relationship. In which context, mixed findings were actually documented regarding the inhibitory effect of fixed mindset on the motivational aspects of creativity (e.g., Zhou et al., 2020; Yeh et al., 2023). This study, by assessing creativity motivation using a standardized measure (i.e., the Creativity Motivation Scale; Zhang et al., 2018) that conceptualizes creativity motivation as a dynamic, developmental process that includes doing, learning, and accomplishing new things, took an initial step by presenting direct and alternative empirical evidence to illustrate the partial mediating role of creativity motivation in the negative effect of fixed creative mindset on reduced creative thinking. Moreover our findings further enrich the mindset-motivation-creativity literature by presenting additional empirical evidence regarding the strength of impact of the motivational mechanism (i.e., 18.8%) that underlies the negative link between fixed creative mindset and creative thinking. However, by taking into consideration that the findings with respect to the inhibitory effect of fixed mindset on the motivational aspects of creativity are still limited and inconsistent, the findings should be interpreted with caution and further empirical scrutiny is warranted to better illuminate the motivational mechanism of fixed mindset effect on creative outcomes.

### 4.2 Theoretical implications

The findings concerning the significant partial mediating role of creativity motivation with regard to the effects of both the growth and fixed creative mindsets may have theoretical implications. These findings lent empirical support to both social cognitive theory (Bandura, 1997) and mindset theory (Dweck, 1999, 2006) with respect to the motivational mechanism that underlies the impacts of different types of creative mindsets on creative outcomes. The findings thus obtained suggest that an integration of these two theoretical perspectives could serve as a useful theoretical framework for scientific research on the underlying psychological mechanisms that may explain the effect of implicit theories on outcome behaviors. In this connection, it is interesting to note that the mindset effect on performance outcomes is still a debatable issue in the literature. For example, recent meta-analytic studies (Sisk et al., 2018; Macnamara and Burgoyne, 2023) on the effect of growth mindset interventions on academic achievement reported mixed results, suggesting inconclusive findings and causing researchers (e.g., Sisk et al., 2018) to speculate that growth mindset interventions might not be as effective in contributing to academic success as expected by mindset theory (Dweck, 1986, 2016). Macnamara and Burgoyne (2023) also contended that these inconsistent results might be related to bias in study designs and flaws in the research methods employed. The results of this study suggest that it is warranted for further empirical scrutiny to verify the validity and generalizability of the joint effects of mindset beliefs and the underlying motivational mechanism with regard to functioning outcomes in alternative behavioral domains.

The findings concerning the fact that the mediating effect of creativity motivation was stronger for growth creative mindset than for fixed creative mindset are also worthy of attention. Previous research (e.g., Li et al., 2021) that has examined the motivational mechanism underlying the mindset-creativity relationship directly have focused solely on the role of growth creative mindset in this context. The present research, by directly analyzing the mediating role of creativity motivation in the impacts of both the growth and fixed creative mindsets on creative thinking in a single study sample, generated comparative results that indicate that creativity motivation may have a stronger mediating impact on the effect of the growth creative mindset on creative thinking (33.3%) than on that of the fixed creative mindset (18.8%). These findings may make important theoretical contributions with respect to our understanding of the construct structure of creative mindset. In the mindset literature, the question of whether the growth and fixed creative mindsets are two independent constructs or two opposites on the same continuum remains debatable (Hass et al., 2016; Karwowski et al., 2020). The findings indicating different extents of accountability with regard to the impacts of the same mediator (i.e., creativity motivation) on both the growth and fixed creative mindsets with respect to identical samples offer new evidence suggesting that the growth and fixed creative mindsets are likely to be two independent constructs. These findings suggest that it is likely that creativity motivation is one mechanism through which growth creative mindset and fixed creative mindset may contribute to creativity independently through different routes, in different directions, and to different degrees. The findings also suggest that it is plausible that creativity motivation may work together with different sets of other mediating variables and/or moderating variables to influence the impacts of either growth or fixed mindsets on creative thinking. Future research is necessary to investigate these speculative ideas.

### 4.3 Educational implications

The findings of the present research also have important educational implications. Researchers and educators have long highlighted the critical role of creativity in personal achievement and societal development (e.g., He, 2023b; Sternberg et al., 2024). In this context, the value of creativity education in practice has been increasingly recognized (Hui et al., 2019; Li et al., 2024). The results of this study suggest that non-cognitive factors such as growth creative mindset, fixed creative mindset and creativity motivation could have important influence on students’ creative thinking. These findings may imply that non-cognitive aspects of creativity (e.g., growth creative mindset and creativity motivation) can be promising components that should be included in creativity interventions in educational settings. This suggested implication is important when referring to the findings of a comprehensive review study conducted by Sidek et al. (2020), which systematically examined the pedagogical approaches that aimed to foster scientific creativity in educational settings and illustrated that although all of the interventions were found to have positive effects on nurturing students’ creativity, few studies focused on the non-cognitive aspects of creativity (e.g., attitude and motivation). The findings of the present study encourage an inclusion of non-cognitive components into a creativity intervention program for students.

In addition to pedagogical approaches, the implementation of growth mindset interventions in school is becoming increasingly popular. Many resources have been allocated to growth mindset intervention studies (Macnamara and Burgoyne, 2023). However, the results of the abovementioned meta-analyses (i.e., Sisk et al., 2018; Macnamara and Burgoyne, 2023) suggested that these resources may have been mislocated. The results of the mediation analyses in this study suggest that, in contexts feature creativity motivation, the total effect of growth creative mindset on creative potential is amplified. These findings may imply that students may benefit more from their creative potential if, alongside the implementation of interventions related to growth creative mindset, educators can enhance students’ creativity motivation.

Moreover, the mindset beliefs that teachers bring into the classroom shape their educational practices (Nelson and Yang, 2023), and further studies can also be conducted to determine whether the findings of this study, i.e., creativity motivation may mediate the relationship between creative mindsets and creativity, can be extended to teaching practices. Although limited studies have investigated such mindsets as well as creative teachers and creative teaching, some previous studies have reported results similar to those of this study. For instance, Nalipay et al. (2021) indicated that growth mindset positively predicted autonomous motivation, which in turn predicted higher work engagement. Fleet and Dobson (2023), based on their exploration of teachers’ creative mindsets, suggested that to facilitate students’ development of creative thinking skills, teachers’ professional development must develop communities of creative practice in the entire school that can focus on actively exploring various conceptualizations of creativity. Hence, teaching preparation and professional development may need to nurture teachers’ growth creative mindset and creativity motivation to enable them to practice and cultivate creativity in classrooms.

### 4.4 Limitations and directions for future research

Several limitations of the study should be noted with regard to the interpretation of the results. First, a convenience sampling method was used and all participants involved in this study were Chinese university students in Hong Kong. A reliance on a convenience sample of college students from Hong Kong has the limitation to provide a diverse demographic representation, which potentially limits the generalizability of the findings to other populations such as various age groups (e.g., young children and teenagers) or participants with different ethnic and socioeconomic backgrounds. Future research should recruit other types of participants to test the generalizability of the results to different participant populations. Second, measurement constraints could be another concern because the study primarily employed standardized, self-reported measures, which are susceptible to biases like social desirability or self-assessment inaccuracies. In future studies, neuroscience methods (e.g., EEG, fMRI, and ERP) could be alternative options to verify the findings of the current research as recent research has shown that an integration of neuroscientific and behavioral data allows a more objective and comprehensive understanding of the mindset effect (Ng, 2018). Moreover, with respect to the assessment of creative potential, only a single measurement of creativity (i.e., TCT–DP) was used. While creativity researchers have increasingly highlighted the importance of employing a multiple-measurement approach to assess creativity (He, 2023b), future research should use various types of creativity tests (e.g., divergent thinking test, creative problem-solving test, and self-reports of creative accomplishments) with the aim of testing the generalizability of the research findings obtained by the current study.

Third, only a limited set of personal beliefs (i.e., growth creative mindset and fixed creative mindset) were investigated in this study, and creativity motivation was found to be one mechanism through which the growth and fixed creative mindsets were related to creative thinking in a positive and negative manner, respectively. In future studies, more elements of the personal belief system according to social cognitive theory (Bandura, 1997) can be investigated to improve our understanding of how the elements of the personal belief system operate and how the personal belief system as a whole functions to affect individuals’ creative thinking. Moreover, in the implicit theories of creativity, creative mindset is one set of creative beliefs, while creative self-efficacy is another important set of creative beliefs. It was found that creative self-efficacy was more powerful in predicting domain-specific performance than in predicting domain-general performance (e.g., Lu et al., 2023). It may be that creative mindset are also domain-specific. It is interesting to address the domain-specific or domain-general issues in future mindset studies. It would be better to have other measures that have been used in previous studies of creative beliefs and meta-cognitions. For example, creative self-efficacy and creative mindset may overlap with personality variables. Because the Big Five personality factors are generally found to be more heritable and less malleable than meta-cognitive traits, it is important that tests of personality be given in case they are precursors, causal, or related in indirect but important ways to these meta cognitive variables (Karwowski and Lebuda, 2016). Lastly, the use of a cross-sectional design restricts the ability to draw causal inferences between creative mindsets, creativity motivation, and creative potential. Future studies should collect longitudinal data to verify the hypothesized links.

## 5 Conclusion

These limitations notwithstanding, the present study makes important contributions to the literature by uncovering the mediating role of creativity motivation in the effects of both the growth and fixed creative mindsets on creative thinking. The findings thus obtained lent empirical support to the perspectives of social cognitive theory and mindset theory by highlighting the critical influences of personal factors such as creativity beliefs and creativity motivation on creative outcomes. The findings further enrich the discourse of social cognitive theory and mindset theory by demonstrating that creativity motivation has a stronger mediating effect on the impact of growth creative mindset on creative thinking than on that of fixed creative mindset on creative thinking. The findings shed important light on effective pedagogical approaches, intervention strategies, and teacher development in the context of creativity education.

## Data availability statement

The original contributions presented in the study are included in the article/supplementary material, further inquiries can be directed to the corresponding author.

## Ethics statement

The studies involving humans were approved by The Human Research Ethics Committee of the Education University of Hong Kong. The studies were conducted in accordance with the local legislation and institutional requirements. The participants provided their written informed consent to participate in this study.

## Author contributions

W-jH: Writing – review & editing, Writing – original draft, Supervision, Methodology, Investigation, Funding acquisition, Formal analysis, Data curation, Conceptualization. T-wC: Writing – original draft, Conceptualization.
